# A computational model of the human glucose-insulin regulatory system^☆^

**DOI:** 10.1016/S1674-8301(10)60048-6

**Published:** 2010-09

**Authors:** Keh-Dong Shiang, Fouad Kandeel

**Affiliations:** a Division of Biostatistics, Department of Information Sciences, City of Hope National Medical Center, Duarte, CA 91010-3000, USA; b Division of Hematopoietic Stem Cell and Leukemia Research, City of Hope National Medical Center, Duarte, CA 91010-3000, USA; c Division of Diabetes, Endocrinology and Metabolism, City of Hope National Medical Center, Duarte, CA 91010-3000, USA

**Keywords:** Coupled ordinary differential equations, glucose tolerance test, parameters estimation, sum of squared residuals, cost function, multiple shooting method

## Abstract

**Objective:**

A computational model of insulin secretion and glucose metabolism for assisting the diagnosis of diabetes mellitus in clinical research is introduced. The proposed method for the estimation of parameters for a system of ordinary differential equations (ODEs) that represent the time course of plasma glucose and insulin concentrations during glucose tolerance test (GTT) in physiological studies is presented. The aim of this study was to explore how to interpret those laboratory glucose and insulin data as well as enhance the Ackerman mathematical model.

**Methods:**

Parameters estimation for a system of ODEs was performed by minimizing the sum of squared residuals (SSR) function, which quantifies the difference between theoretical model predictions and GTT's experimental observations. Our proposed perturbation search and multiple-shooting methods were applied during the estimating process.

**Results:**

Based on the Ackerman's published data, we estimated the key parameters by applying R-based iterative computer programs. As a result, the theoretically simulated curves perfectly matched the experimental data points. Our model showed that the estimated parameters, computed frequency and period values, were proven a good indicator of diabetes.

**Conclusion:**

The present paper introduces a computational algorithm to biomedical problems, particularly to endocrinology and metabolism fields, which involves two coupled differential equations with four parameters describing the glucose-insulin regulatory system that Ackerman proposed earlier. The enhanced approach may provide clinicians in endocrinology and metabolism field insight into the transition nature of human metabolic mechanism from normal to impaired glucose tolerance.

## INTRODUCTION

Diabetes mellitus is a chronic metabolic disorder characterized by abnormally high urine and blood glucose levels (i.e., hyperglycemia) due to insufficient insulin levels. Based on the statistics of American Diabetes Association (ADA)[1], approximately 23.6 million people, or 7.8% of the population, in the USA are afflicted with this disease. While an estimated 17.9 million people have been diagnosed with diabetes, regrettably, 5.7 million people (or nearly one quarter) are unaware that they have the disease. The total annual economic cost of diabetes in 2007 was estimated to be $174 billion. Diabetes can cause serious health complications including blindness, heart disease, kidney failure, stroke, nerve damage, and lower extremity amputations. According to the statistical report from Centers for Disease Control and Prevention (CDC), di-abetes is the sixth leading cause of death in the USA[2]. Consequently, diagnosis, treatment, control and prevention of diabetes, are extremely critical in the current medical era.

In a normal subject, the beta-cells (*β*-cell) in the pancreas release insulin in response to rises in the level of glucose in the blood, which results in the storage of this source of energy as glycogen in the liver. Type I diabetes, also called juvenile or insulin-dependent diabetes mellitus (IDDM), often manifests in childhood and may result from autoimmune destruction of insulin-producing *β*-cells of the pancreas. Thus, insulin hormone can no longer be produced. This type of diabetes is fatal without treatment with exogenous insulin to replace the missing hormone or providing patients with a functional replacement for the destroyed pancreatic *β*-beta cells, such as pancreas or islet-cell transplantation. Type II diabetes (formerly called non-insulin-dependent diabetes mellitus, NIDDM, or adult-onset diabetes), a more widespread metabolic disorder, is primarily characterized by insulin resistance, relative insulin deficiency and hyperglycemia. Some cases of type II diabetes also appear to be an autoimmune disease where the immune system attacks the *β*-cells, decreasing the function of producing insulin, while other type II diabetes cases may simply result from excessive body weight that strains the ability of the *β*-cells to produce sufficient insulin. However, in both type I and type II cases, the human body loses its ability to regulate blood sugar, which causes a significantly negative effect on the patients' quality of life or even be potentially fatal.

It is a common knowledge that blood glucose concentration in normal humans is maintained within a precise and stable range. Many external and internal factors affect the level of blood glucose such as food intake, rate of digestion, excretion, exercise, sleep, and psychological state. These individual or combinational influences constantly alter the physiological processes that regulate plasma glucose level. For instance, if blood glucose is elevated, after a regular meal (i.e., post-prandial), certain cells in the pancreatic islets of Langerhans named *β*-cells, will release the insulin hormone. The secreted insulin, then, leads to the uptake of glucose from the blood into the liver and other cells, such as muscle cells. Thus, blood glucose level will eventually go down to the normal range. On the other hand, blood glucose level may decrease imminently due to muscular activity, particularly when food intake is confined. This reduced level of blood glucose is immediately recognized by other sensitive pancreatic cells, the alpha-cells (α-cells). These cells then release glucagon that act on the cells of the liver to initiate the release of glucose. This results in blood glucose level elevating back to the normal range. Briefly, these islet-cell arguments establish the fact that the capacity to lower blood glucose depends on the responsiveness of the pancreatic beta-cells to glucose and the sensitivity of the glucose utilized by tissues to the released insulin. Thus, both pancreatic *β*-cell responsiveness and insulin sensitivity contribute to glucose tolerance. Low glucose tolerance in lean humans could be associated with diminished *β*-cell response to glucose, whereas low glucose tolerance in obese people could be associated with decreased insulin sensitivity. Furthermore, a shortage of plasma insulin and low glucose tolerance, resulting in a serious inability to lower blood glucose, will cause insulin resistance, which is the key symptom underlying the potential development of diabetes. However, to tackle diabetes disease and obesity problems, clinicians and researchers are now turning to mechanism-based mathematical models to reach quantitative diagnoses of glucose intolerance and insulin resistance, and also to predict the likely outcomes of therapeutic interventions. Their ultimate goal is to develop a mathematical model that can be used to accurately predict the outcomes and most successful treatment options for people who have diabetes.

Speaking of theoretical solutions to diabetic problems, we should mention the term, “mathematical model”, which is a representative depiction of a real system *via* mathematical tools in these medicines' golden years. The fundamental nature of a good mathematical model must be simple in design and exhibit the basic properties of the real system that we are attempting to simulate and understand. All well-developed models should be validated and tested against empirical data. In a practical sense, the quantitative comparisons of the model to the real system should lead to an improved mathematical model. The successful model can be applied to suggest the corresponding experiment to highlight a particular aspect of the weakness or problem, which may improve the method of data collection or the procedure of experimental processes. Thus, modeling itself is an evolutionary process, which is a evolving procedure in which something changes into a different but better form. Similarly, developing and using a successful mathematical model will guide us to learn more about certain simulating or existing processes rather than finding an entirely actual state of the system.

During the last few decades, a massive range of mathematical models, computer algorithms and statistical methods have been proposed in order to understand different aspects of diabetes, such as glucose metabolism, insulin kinetics, *β*-cell mass, and the glucose-insulin regulatory system. Several reviews have been devoted to mathematical models and diabetic disease[3]–[8] and are worthwhile to be referenced. Other than those reviewing journal articles, a pioneering work on modeling the glucose-insulin regulatory system and its ultradian insulin secretory oscillations can be traced back to Bolie[9]. In this pioneering study, a system of glucose-insulin regulation in terms of coupled differential equations of feedback was analyzed with the so-called critical damping criteria of a self-regulating feedback system (i.e., servomechanism theory). The secretion of insulin in the glucose-insulin endocrine metabolic system occurs in an oscillatory manner over a range of 50-150 min and is usually referred to as ultradian oscillations[10]. In 1965 and 1969, Ackerman *et al.*[11],[12] pioneered the concept of offering their full exploratory work for the blood glucose regulatory response to the glucose tolerance test (GTT), which was governed by two coupled differential equations. In the following sections, we will introduce their conceptually illuminating model in greater detail, and also develop our computational model, which will be validated by using their model equations and other published experimental data and results.

In order to determine whether or not a patient has pre-diabetes or diabetes, health care providers usually conduct a fasting plasma glucose (FPG) test or a GTT. The ADA recommends the FPG test because it is easier, faster, and less expensive. Therefore, in the following, several quantitative assessment methods are briefly introduced. Unquestionably, due to the advantage of skipping GTT procedures, fasting plasma glucose level is simpler and quicker to measure, and its measurement is more acceptable to patients than any glucose tolerance test.

The homeostasis model assessment (HOMA) is an index of insulin resistance (IR). It was developed by Matthews *et al.*[13],[14] and derived from the product of FPG and fasting plasma insulin (FPI) divided by a constant, 22.5.


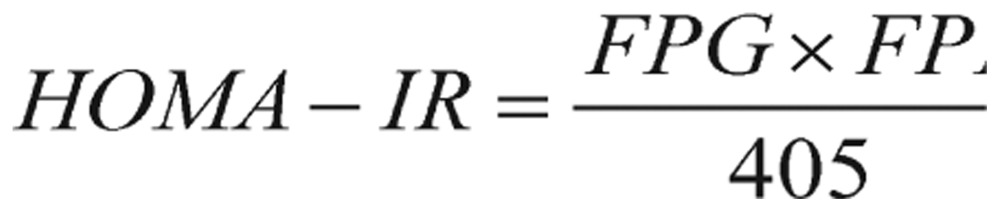
(1.1)

where glucose is given in mg/dL and insulin is given in µU/mL. In this equation, the constant 405 should be replaced by 22.5 if the glucose is reported in mmol/L.

Since hepatic glucose production (HGP) is the main determinant of FPG concentration, and FPI concentration is the main regulator of HGP, HOMA-IR index is practically a measure of hepatic IR. For easy interpretation, lower HOMA-IR values indicate greater insulin sensitivity, whereas higher HOMA-IR values indicate lower insulin sensitivity (i.e., IR).

Another way to see this HOMA index function, another index, insulin sensitivity (IS), is defined as 
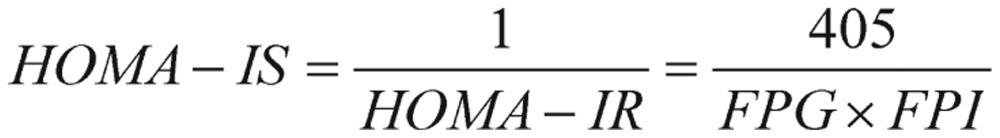
(1.2)

By applying the same fasting values, pancreatic *β*-cell function (HOMA *β*-cell) can be estimated by the evaluation form: 
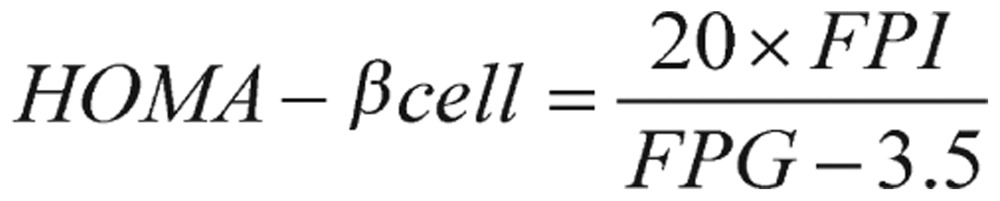
(1.3)

where the unit of FPI is µU/mL, and the unit of FPG is mmol/L.

Moreover, a worth mentioning index, Quantitative Insulin Sensitivity Check Index (QUICKI)[15], is derived by calculating the inverse of the sum of logarithmically expressed values of fasting glucose and fasting insulin: 
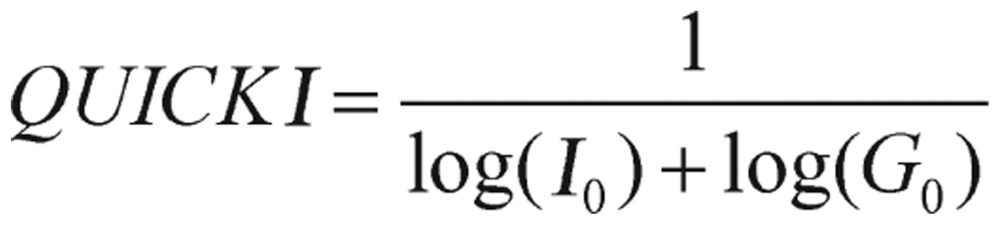
(1.4) in which G_0_ is the fasting glucose level and I_0_ is the fasting insulin value. Many investigators and researchers believe that QUICKI is superior to HOMA in determining insulin sensitivity, although those two values correlate well.

Other than the above fasting-value methods, the simplest and widely used test for detection of diabetes is GTT. For this test, a subject fasts for 12 h, and is then given a large amount of glucose. During the next few hours, blood samples are drawn and glucose levels are measured and recorded. By fitting the GTT data to a mathematical model proposed by Ackerman *et al.*[11],[12], the diagnosis information from the model can be applied to indicate which subject has diabetes. This model is described by a differential equation system where the variables are deviations of glucose levels from the subject's baseline value in blood (in the morning after fasting overnight) and the similar deviation of insulin concentration. The differential equation system governing this GTT model is expressed as follows: 
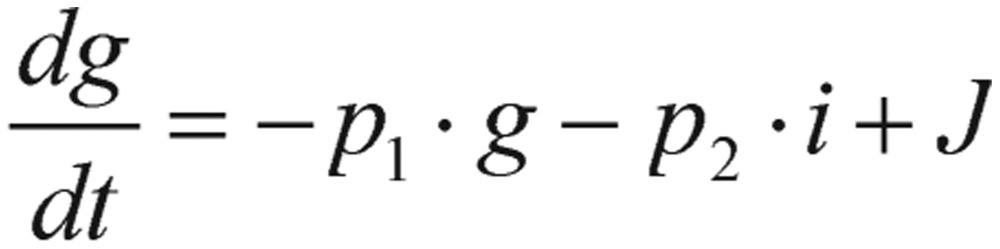
(1.5)

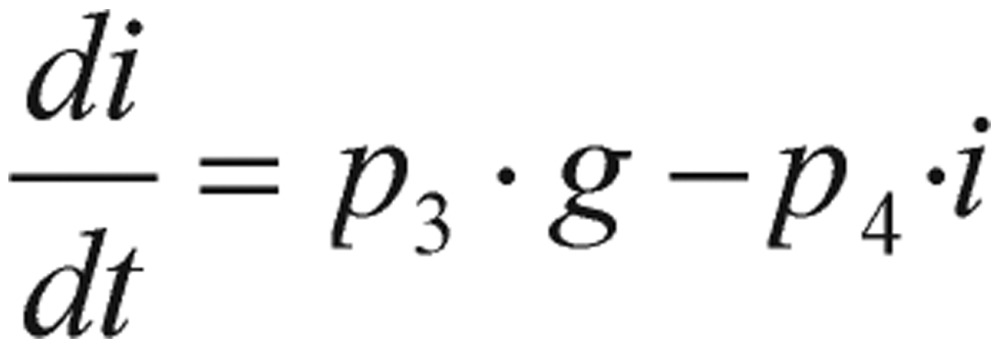
(1.6) where *p_i_* (*i*=*1, 2, 3, 4*), are positive constants, *J* is the rate of glucose infusion from the intestines (or intravenously), *g(t)* is the difference between blood glucose concentration *G(t)* and its baseline value *G_0_*, and *i(t)* is the difference between plasma insulin concentration *I(t)* and its baseline value *I_0_*, as shown in the following equations.


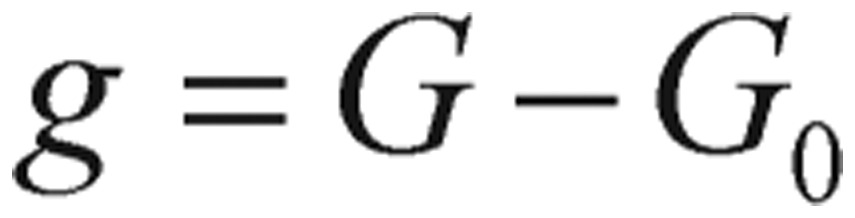
(1.7)


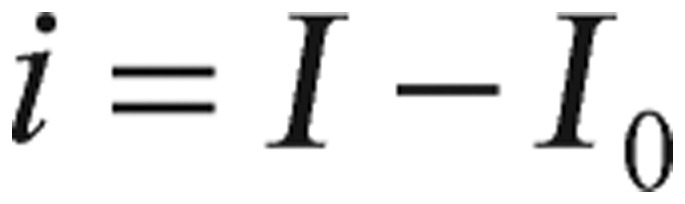
(1.8)

**Fig. 1:**
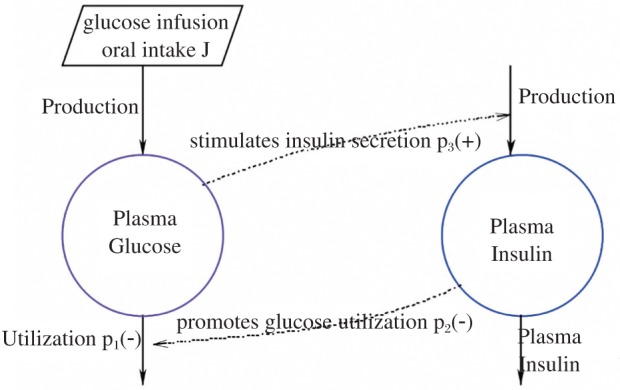
Sketchy diagram of the mathematical model of the glucose-insulin regulatory system.

The reason for this variable transformation is because we are usually more interested in the difference values (i.e., fluctuations or excursions) of glucose and insulin (i.e., the relative values, not the absolute values). The diagram of this two-compartment model is shown in ***Fig. 1***.

The construction of the model equations (1.5) and (1.6) is based on the following hypotheses:

1) Each variable, *g* and *i*, has various influences upon the appropriate change speed with a negative feedback (i.e., utilization or clearance) process, which is shown as the parameters -*p_1_* and -*p_4_* in both equations.

2) An increase in blood gluose levels provokes an increase of insulin secretion, which is expressed as the positive feedback (i.e., stimulation) parameter *+p_3_* in the second equation.

3) An increase of hormone insulin secretion leads to a reduction in blood glucose levels, which is formulated as the negative feedback (i.e., utilization) parameter-*p_2_* in the first equation.

The differential term *dg/dt* is defined as the change in blood glucose difference with respect to the change in time. Similarly, the expression *di/dt* is defined as the change in plasma insulin difference with respect to the change in time. To illustrate this method, we have simulated the glucose-insulin dynamics system for both normal and diabetic subjects in the Results section. For the purpose of algorithm integration, the section “Computational methods and theory” will describe the mathematical formulation in greater detail. It has to be noted here that the analytical demonstration of an appropriate qualitative and quantitative behavior for this mathematical model is the great departure point for the subsequent experimentally clinical and theoretically numerical determination of an optimal key parameter for diagnosing the disease.

It has been reported that glycemic variability (or brittleness) scores can be assessed by both the Lability Index (LI)[16],[17] and the Mean Amplitude of Glycemic Excursions (MAGE)[18]–[20] methods.

This LI score provides a measure of blood glucose variability in diabetes and is based on the square of change in levels of blood glucose from one reading to the next, divided by the time interval and summed for a week. A value of LI for each of 4 w is derived based on computing the following sum for each week in the period: 
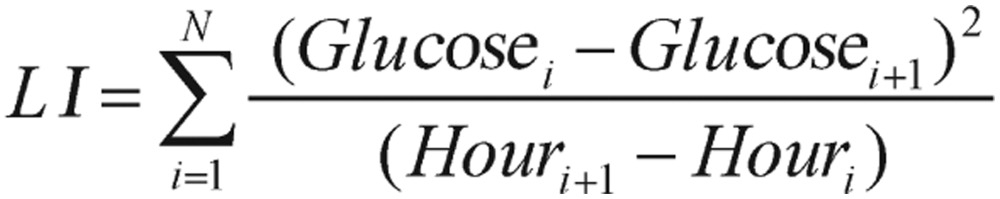
(1.9)

where glucose (mmol/L) is the *ith* reading of the week taken at time *Hour_i_*. The upper bound, *N*, is the total number of readings in 1 w. The minimum and maximum time intervals used are 1 h and 12 h, respectively. Consequently, the median LI in type I diabetes control subjects (*n* = 100) was 223 m(mol/L)^2^/(h·week) (25^th^ to 75^th^ interquartile range: 130-329). Patients who received islet transplant (*n* = 51) had a median LI value of 497 m(mol/L)^2^/(h·week) (25^th^ to 75^th^ interquartile range: 330-692) before transplantation. After transplantation, their median LI values became 40 m(mol/L)^2^h·week (25^th^ to 75^th^ interquartile range: 14-83). It is clearly seen that there is a large decrease in LI scores after islet transplantation. This indicates that islet transplantation is effective in curing type I diabetes and also results in better glucose control (i.e., smaller blood glucose swings) for these patients, at least, for the first few years after their transplantations. It is also shown that the LI scoring system does provide a metric to make a quantitative comparison between patient groups and also complement the clinical assessment of glucose variability in diabetic patients.

The MAGE is another measurement and a reflection of how much blood glucose increases or decreases throughout a day. It measures the amplitude of the daily “large” blood glucose excursions. The question is “how large is the amplitude?” The answer is that the intraday glycemic excursions with their amplitudes are greater than one standard deviation (SD). Theoretically, the MAGE requires at least 14 blood glucose measurements over consecutive 48 h before and 2-h after breakfast, lunch and dinner, and at bedtime with an optional measurement at 3 AM. A glycemic excursion is then computed as the absolute difference in peak and subsequent nadir (or *vice versa*) glucose values, with the direction (decrease - peak to nadir *vs* increase - nadir to peak) determined by the first quantifiable excursion in the 48 h. All excursions greater than one SD of the 7+ glucose readings for the day were summed and divided by the number of qualified excursions to give the MAGE score in mg/dL (or mmol/L) glucose. To emphasize major glucose swings and eliminate minor ones, however, excursions less than one SD are ignored. It is recognized that the MAGE scores are lower in healthy research participants than in diabetic research participants. In other words, the lower the MAGE score, the less severe the swings of the blood glucose levels. Roughly speaking, MAGE score average is approximately about < 90 mg/dL in healthy subjects and about > 150 mg/dL in diabetic patients or subjects with poor glycemic control. As shown in the study by Ryan *et al.*[16],[17], blood glucose excursions expressed as MAGE score were significantly lower after islet transplantation.

A well-known blood glucose meter, continuous glucose monitoring system (CGMS), is an FDA-approved device that records subjects' blood glucose levels throughout the day and night. In other words, CGMS is used to provide continuous “real-time” readings about trends in blood glucose levels[21]. This may allow users to know the levels of their glucose and whether they are rising or falling and to intervene by eating food or taking insulin to prevent them from going too high or too low. Clinically, blood glucose regulation can be evaluated based on the CGMS device. DirecNet study group[22] has pointed out that no simple test currently exists for assessment of glycemic variability in patients with diabetes. They have done the first report to compare eight-point testing with CGMS as a means to evaluate glycemic control. Despite the much larger number of measurements with CGMS than with eight-point testing, the overall mean glucose levels were nearly identical. However, these few-sample-point device and CGMS are the essential tools in measuring the blood glucose levels, and the recorded data are used for physiologic analysis to control the glucose variance.

**Fig. 2:**
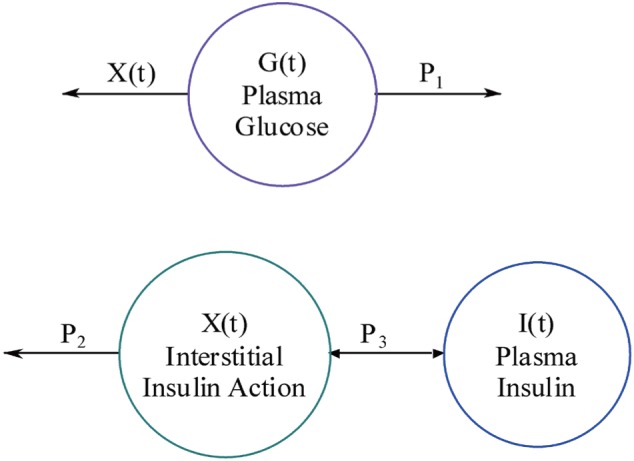
The compartmental flow chart of the minimal model for glucose kinetics.

Recently, the Minimal Model (MM), proposed by the team of Bergman and Cobelli[23]–[35], is one of the most informative computational methods for studying glucose and insulin kinetics in metabolism. The MM for glucose kinetics is illustrated in ***Fig. 2***.

In this figure, *I(t)* is the plasma insulin level, and *I_b_* represents its basal level; *G(t)* is the plasma glucose level, and its basal level is denoted as *G_b_*. The coupled differential equations corresponding to the glucose minimal model are expressed as 
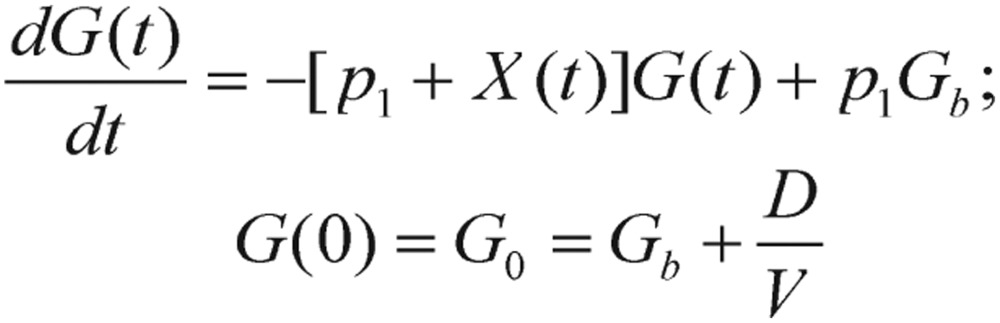
(1.10)

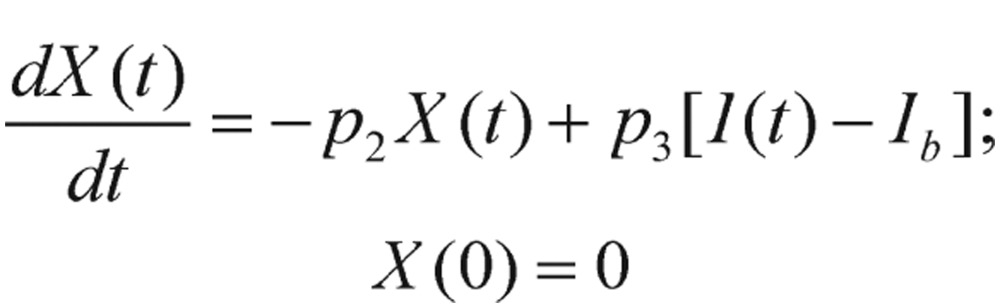
(1.11)

with *G(0)=G_0_* and *X(0)=0*. In these equations, *X(t)* is the interstitial insulin at time *t*. There are a total of four unknown parameters in this model: *G_0_, p_1_, p_2_*, and *p_3_*, which are also defined with the units and brief descriptions are provided below:*p_1_*[min^−1^], glucose effectiveness, *p_1_=S_G_*, the rate of net glucose utilization without dynamic insulin response (i.e., insulin-independent).*p_2_*[min^−1^], rate constant expressing the spontaneous decrease of tissue glucose uptake ability.*p_3_*[min^−2^ (µU/mL)^−1^], insulin-dependent increase in tissue glucose uptake ability.*G_0_*[mg/dL], theoretical glycemia at time 0 after the instantaneous glucose bolus.

The MM describes the time-course of glucose plasma concentrations, depending upon insulin concentrations and the new intermediate variable *X*, representing the “insulin activity in a remote compartment”. This synthetically contrived and physiologically inaccessible variable *X* plays a transitive role between blood glucose and plasma insulin. It is clearly shown that this virtual variable in equation (1.11) has replaced the position of the insulin variable in equation (1.6).

It is commonly recognized that MM is a well-known and successful mathematical model in simulating glucose metabolism and insulin kinetics. This is also why we cannot skip the introduction of the model. In our current study, however, we focused mainly on presenting a new approach of parameters estimation on glucose-insulin regulatory system. Since MM has been extensively studied by many clinicians and researchers, we have restricted ourselves to only the coupled ordinary differential equations based on the Ackerman's model. Unquestionably, the application of MM using our proposed approach will be carried out in future trials.

## COMPUTATIONAL METHODS AND THEORY

### Glucose-insulin dynamics

The majority of mathematical models proposed in the literature were dedicated to the dynamics of glucose-insulin, including oral glucose tolerance test (OGTT), intravenous glucose tolerance test (IVGTT), and frequently sampled intravenous glucose tolerance test (FSIVGTT). In 1970, various mathematical models have been applied to evaluate glucose disappearance and insulin-glucose dynamics[36]–[38]. Particularly, Bolie[9], among the pioneers in this field, used two coupled ordinary differential equations to constitute the following simple model: 
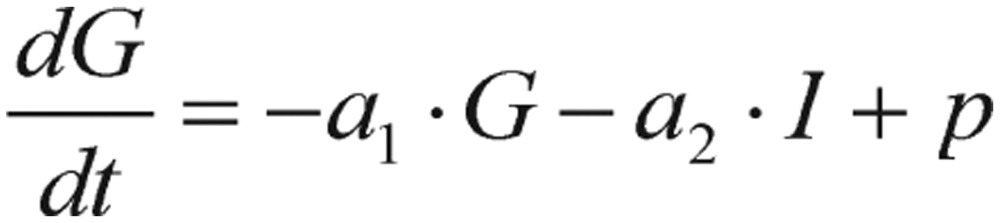
(2.1)

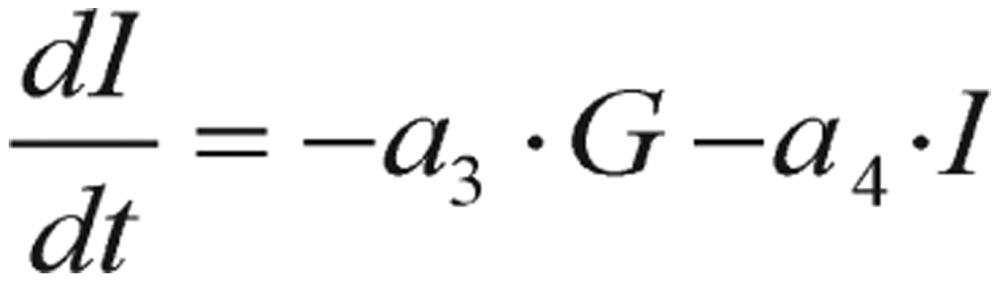
(2.2) where *G = G(t)* represents blood glucose concentration, *I = I(t)* represents plasma insulin and *p*, *a*_1_, *a*_2_, *a*_3_, and *a*_4_ are parameters.

In 2005, Wu[39] proposed the following model for self-management of type II diabetes: 
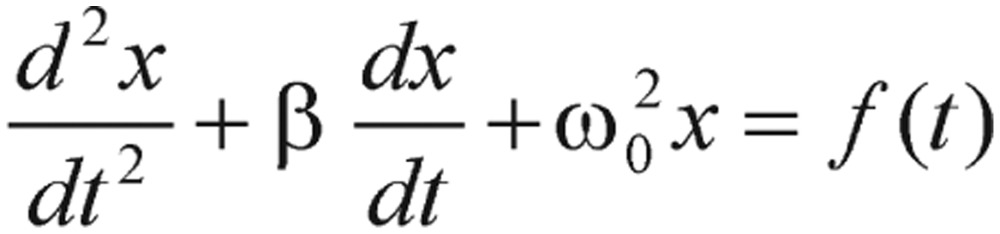
(2.3) where *x* represents blood glucose level over the baseline at time *t* and *ω_0_* is the system natural frequency. The postprandial blood glucose excursion can be considered as a hormone regulated springy system. The food intake is treated as a bolus injection of glucose and expressed as the impulse force *f(t)*. Effects of exercises and hypoglycemic medication are lumped as the damping factor, *β*. The differential equation of such an oscillatory system, which is used to describe postprandial blood glucose excursions, can be found in many physics and mathematics textbooks.

Generally speaking, preprandial blood glucose levels are generally fluctuating with relatively insignificant magnitudes, and, thus, can be approximated as a flat level. If the impulse force *f(t)* takes the form of the Dirac delta function, *F*δ*(t-0)* with *F* being a food intake dependent parameter, the solution of equation (2.3) is given by 
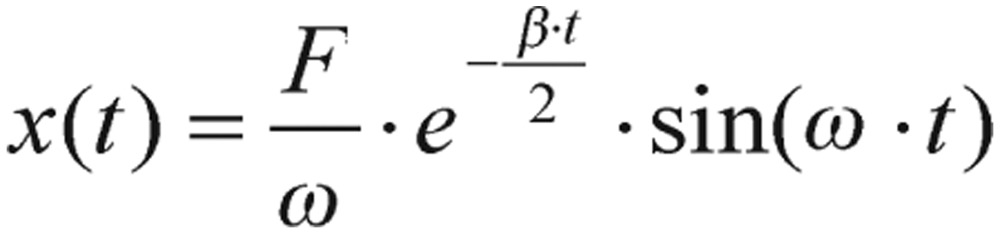
(2.4) where 
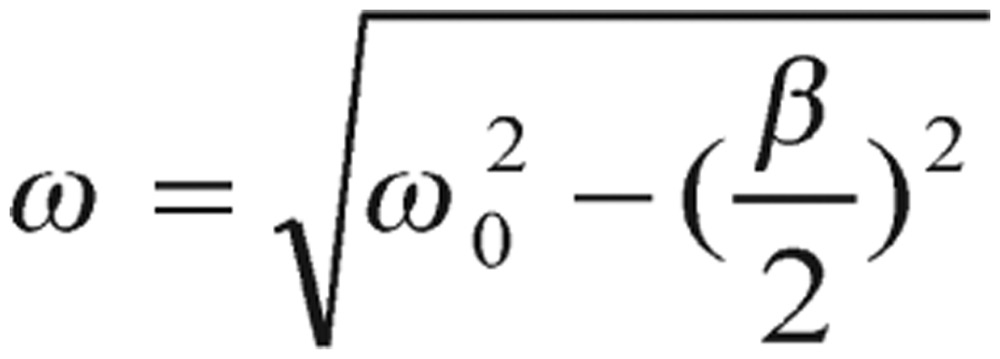
(2.5) is the frequency of the system. As you can see from equation (2.4), it is described as a three-parameter model, *F*, *ω* and *β*. Implications of these three parameters not only could reveal distinctive characteristics of diabetic and normal individuals but also provide guidelines to adjust diabetic patient's treatment and lifestyle.

It has been demonstrated in the study of Ackerman *et al.*[40] that the response of blood-glucose concentration *(G)* as a function of time *(t)* is represented adequately by an equation involving four parameters in the equation: 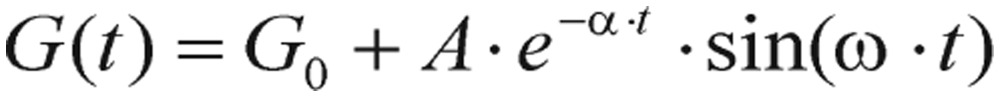
(2.6)

The values of these four parameters are defined by the four experimental measurements (see ***Table 1***) usually made in an ordinary GTT. On the basis of measurements in over 750 persons in their study, it was suggested that the value of *ω_0_* could be used to distinguish normal from diabetic subjects more closely than any other parameters. It should also be noted that the solution of the oscillatory pattern in equation (2.6) has the same form as the expression in equation (2.4).

As indicated earlier, the mathematical models proposed by Ackerman[11],[12],[40], Bolie[9] and Wu[39] are the simplest models and have been indisputably useful in physiological diabetes research and served as a starting point for many other models. Therefore, our present work extends the scope of the public-recognized model by introducing a novel approach using the perturbation method for parameters estimation and the multiple-shooting method for solving two coupled ordinary differential equations on glucose-insulin regulatory system.

The derived model equations in this study are written as the forms in equations (1.5)-(1.8), which describes the blood glucose regulatory system during a GTT. Actually, the starting equations are expressed as these simple forms: 
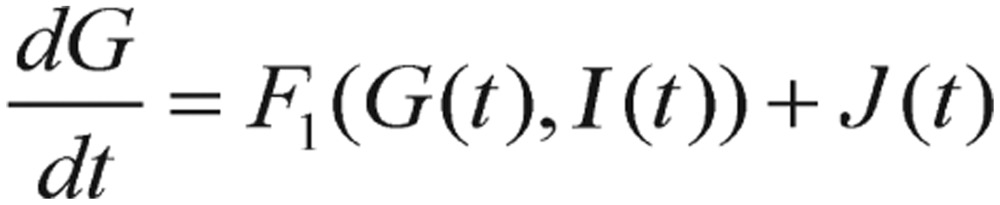
(2.7)

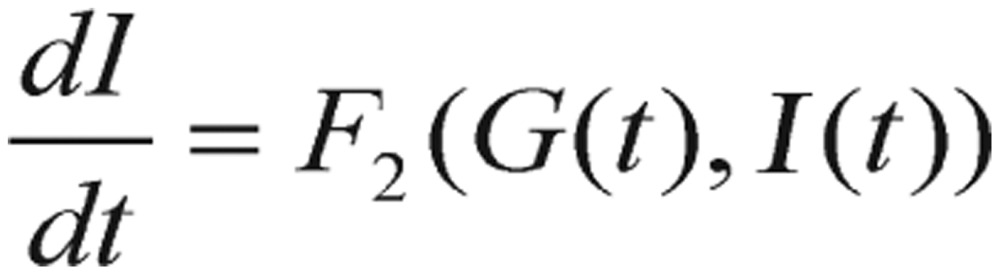
(2.8)

where *t* is time, *G* is blood glucose concentration, and *I* is plasma insulin concentration. The impulse function, *J(t)*, is basically a delta function representing the large dose of glucose given initially in the GTT after the subject has fasted. In this section, we only briefly list some key formulas and equations for the purpose of attracting readers' focus.

After a lengthy derivation, the deviation *g(t)* of a subject's blood glucose concentration satisfies the second-order differential equation: 
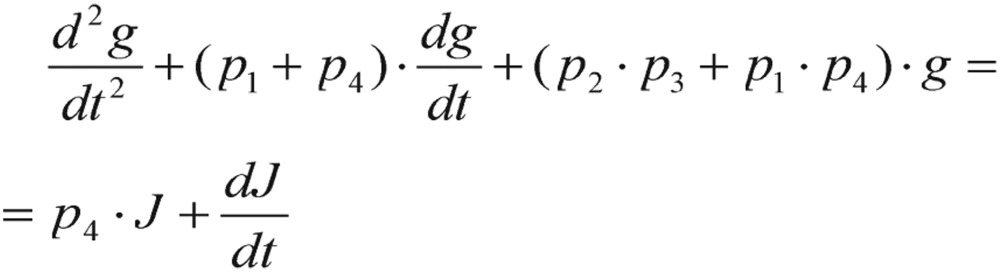
(2.9) which can be rearranged as 
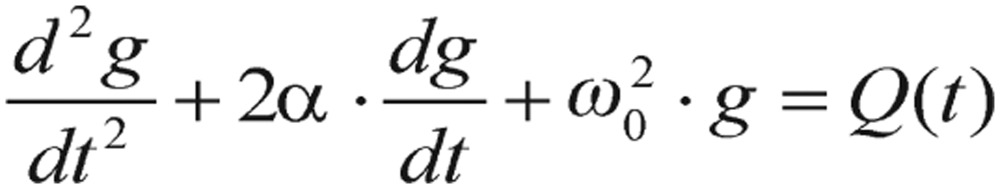
(2.10)

where the decay constant 
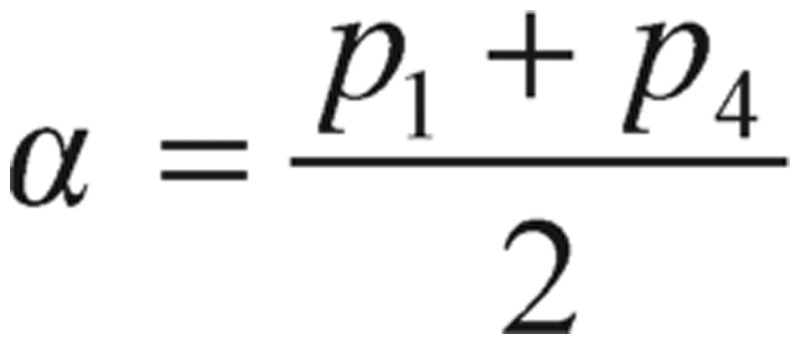
, the natural frequency 
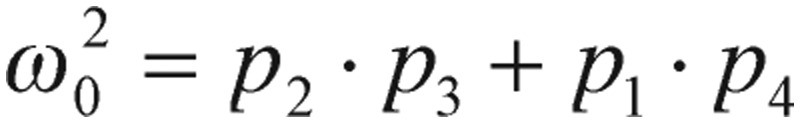
, and the input glucose impulse function 
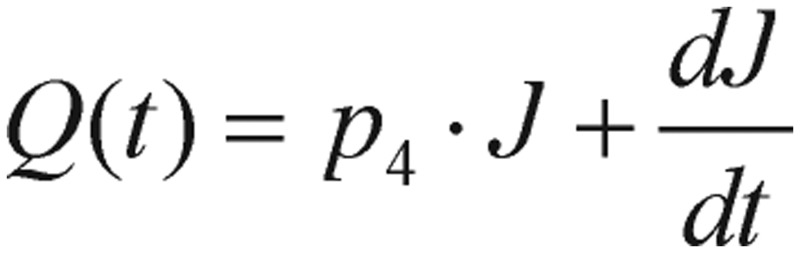
. During a very short period of time at *t* = *0*, the *Q(t)* can be expressed as a Dirac delta, *δ*, function. After starting GTT, *t* > *0*, the *Q(t)* function is assumed to be zero.

In physics terminology, if a frictional (i.e., damping -*α* term) force proportional to the velocity does exist, the harmonic oscillator will be described as a “damped” harmonic oscillator. When the damping factor, *α*, is greater than zero, the dynamic system will decay, but may or may not oscillate, depending on the relation between the damping factor *α* and the natural frequency *ω_0_*. As a consequence, the solution *g(t)* will have three possible outcomes:

1) When 
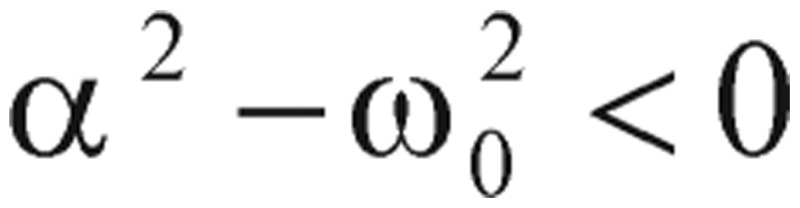
, the solution has an exponential decay with the oscillatory component. This is called the “under-damping” solution.

2) When 
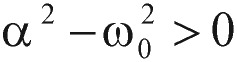
, the solution represents an exponential decay or growth. This is called the “over-damping” solution.

3) When 
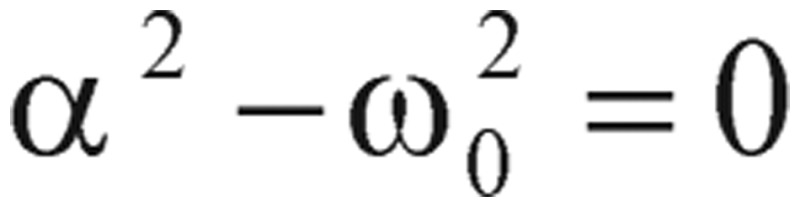
, the “critically damped” oscillator rapidly approaches the maximum position, and then smoothly drops to the equilibrium state.

However, all those curve tendencies may not be reflected as the same patterns on other damped oscillatory systems, as they really depend upon their amplitudes, angular frequencies and damping factors. Consequently, the period of oscillation is finally formulated as 
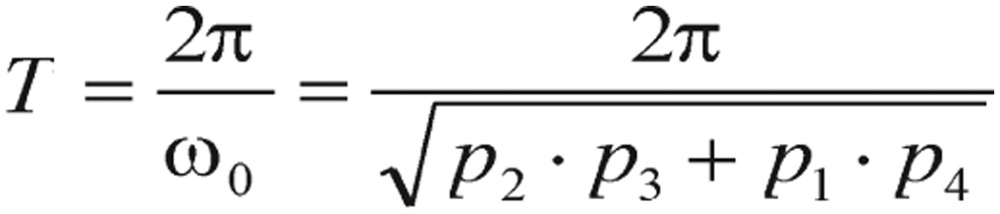
(2.11)

According to the general diabetologist's theory (detailed results and references in section Results), healthy individuals should satisfy the condition of *T*=*2π/ω_0_* < *4 h*, conversely, while the reversed inequality of the period T indicated diabetic cases. As a matter of fact, this conditional situation actually occurs frequently in practice - the blood glucose concentration of a non-diabetic who has just absorbed a large amount of glucose will be at or below fasting level in 2 h or less.

### Nonlinear Least Squares Fitting and Gauss Newton Method

The linear regression model is a system of linear equations that can express the model using data matrix *X*, response vector *Y* and parameter vector *P*. The *i*^th^ row of *X* and *Y* will contain the *x* and *y* value for the *i*^th^ data element. Thus, the model can be written as a detailed matrix form: 
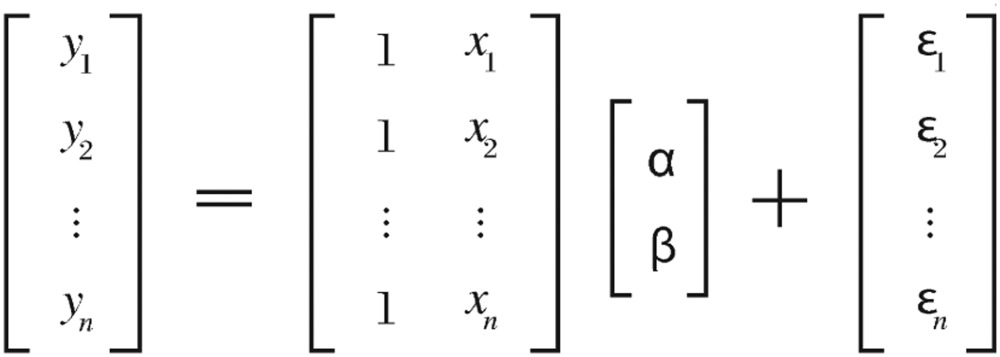
(3.1) where *ϵ* is the normally distributed random error term with the expected value of a column vector of zeros and variance *σ*^2^ *I_n_*, where *I_n_* is the *n*×*n* identity matrix.

The pure matrix notation becomes 
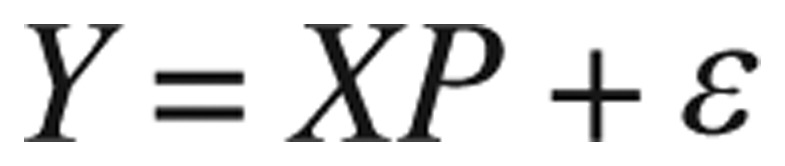
(3.2) where

*Y* is an *n*-*by*-*1* vector of responses

*X* is the *n*-*by*-*m* design matrix for the model, where *m* = 2

*P* is a *m*-*by*-*1* vector of parameters, *α* and *β*, where *m* = 2

*ϵ* is an *n*-*by*-*1* vector of random errors

For nonlinear least squares fitting to a number of unknown equations' parameters, linear least squares fitting could be applied iteratively to a linear form of the function until convergence is achieved. Now we consider the nonlinear models of the form 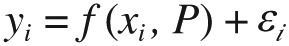
(3.3)

The least squares method still applies, with the sum of squared residuals (*SSR*) function to be minimized with respect to the parameter vector *P*. Please note that *SSR* is sometimes called *RSS* as it stands for residual-squared-sum. The least square estimate of *P*, *Pˆ*, is the set of parameters that minimizes the sum of squared residuals: 
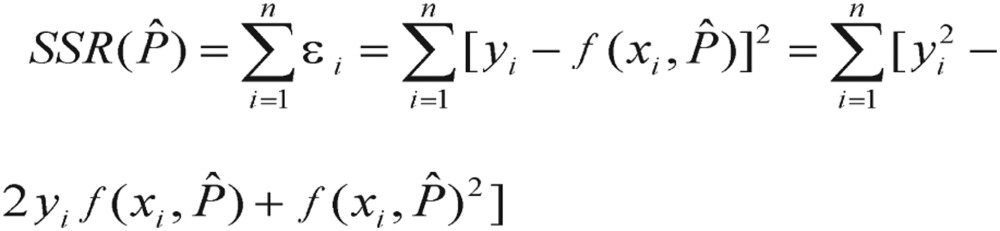
(3.4)

To obtain the normal equations, we consider the partial derivatives of *SSR*(*Pˆ*) with respect to each parameter *Pˆ*, and set them equal to zero. This gives us a system of *m* normal equations. Each normal equation is given by differentiating *SSR*(*Pˆ*), 
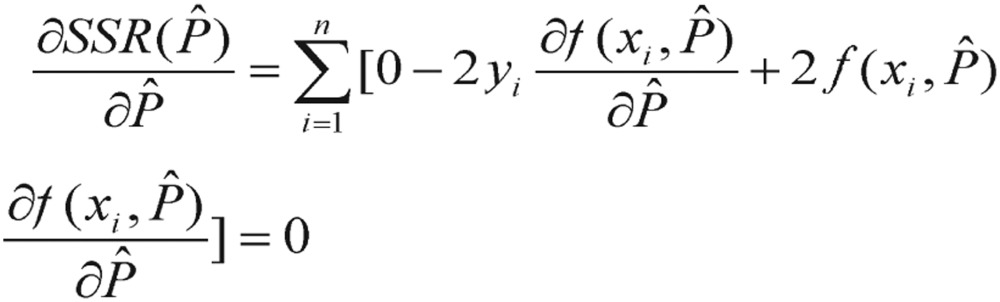
(3.5) It yields 
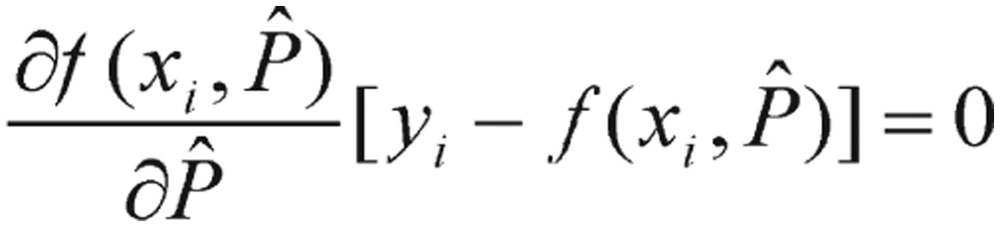
(3.6) Unfortunately, the partial derivatives of nonlinear functions are also functions of the parameters *Pˆ*; thus, an explicit solution (i.e., analytical solution) for *Pˆ* cannot be obtained. We will have to get the solution by numerical optimization.

The basic concept of the Gauss-Newton optimization method can be summarized as:

1) Use a Taylor series expansion to linearize a nonlinear function;

2) Apply least-square theory to obtain new estimate of the parameters that move in the direction of minimizing the *SSR*.

If a continuous function is differentiable in p, it can be linearized locally as 
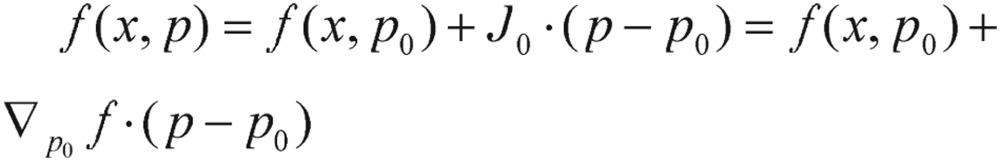
(3.7) where *J_0_* is the n×m gradient matrix with elements 
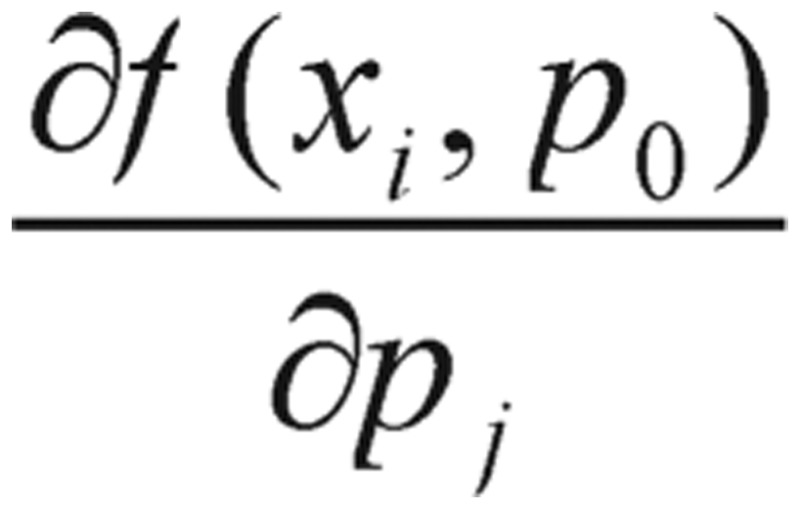
. Here, the number of data points *i* = 1, 2, 3, …, *n*, and the number of unknown equation parameters *j* = 1, 2, 3, …, *m*. This leads to the Gauss-Newton algorithm for estimating the value of *p*, 
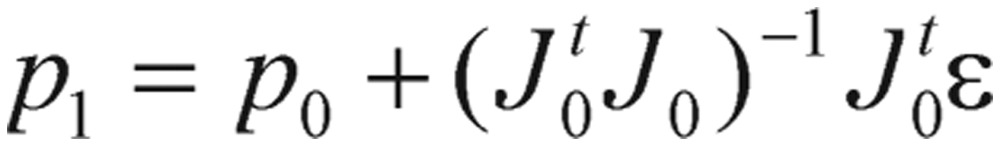
(3.8) where matrix *J*_0_*^t^* is the transpose of matrix *J_0_* and *ϵ* is the vector of residuals. The Gauss-Newton algorithm will converge the estimated parameter values, *Pˆ*, to the solution from a sufficiently good starting value. However, most of the nonlinear regression methods follow these standard steps:

1) Start with initial estimated values for each adjustable parameter in the equations.

2) Generate the data trajectory for the entire curve based on the initial input values. Independent random number generator and numerical integration subroutine are required for implementation.

3) Calculate the sum-of-squares of the vertical distances of the data points from the curve. Or compute the weighted sum-of-squares if weighting factors are included.

4) Adjust the parameters to make the curve come closer to the data points by evaluating the corresponding cost (or objective) function.

5) Stop the calculations and go to step 6 when the adjustments make virtually no difference in the sum-of-squares or any available stopping criteria are satisfied; if not, repeat simulation steps 2-4 with newly selected initial values.

6) Report the best-fitted parameter values.

Please note that steps 2, 3 and 4 are the only ones whose details differ from method to method.

### The Perturbation Search Method

We should remark here that gradient-based methods frequently encounter divergent difficulties, and convergent case only occurs under certain restricted conditions. In addition, gradient-based methods usually pose the risk of being trapped in a local minimum, rather than in the global minimum. If inappropriate values of the initial parameter are selected and run for the entire simulation because they yield the best quality of data fit for the predefined working range, the incorrect conclusion will be drawn quickly without letting developers having any further tests using other initial parameter sets. In common practice, local methods optimize the cost function directly with respect to initial values and parameter vector. This optimization scheme is often called initial-value approach or alternatively named “single-shooting method”. Due to the presence of multiple minima in some cases, convergence of local optimization methods to the global minimum is usually limited to a rather small domain in search space. In contrast, global methods have an essentially larger convergence domain but the computational cost increases rapidly. At this point, it has to be mentioned that one of the stable and effective global searching methods is the multiple-shooting algorithm developed by Bock[41]–[44]; a brief description will be given in the next subsection. The basic idea of the algorithm is to consider the task of the single initial-value approach as a multi-point boundary value problem. Mostly importantly, this multiple-shooting algorithm is used to incorporate the proposed perturbation-search method in our current study. It has been concluded that single-shooting method steps quickly in local minima or runs into a divergent situation, whereas multiple-shooting method performs better than single-shooting approach at the expense of higher computational costs.

As a matter of fact, our concept of the perturbation search method was inspired by the prospect of basic perturbation theory. The principle of perturbation theory is to study dynamical systems that are small perturbations of integrable systems. Perturbation theory is a technique in quantum mechanics for finding approximate solutions to real-world problems. It has a wide variety of applications throughout chemistry, physics and mathematics. In quantum mechanics, we often need to solve the Schrodinger equation that corresponds to a given physical potential or Hamiltonian. For a real-world potential or Hamiltonian, finding a solution analytically is almost impossible. In perturbation theory, a complicated potential is broken down into a solvable potential (without perturbed terms), plus a small-change remainder. Basically, we solve the first part (without perturbed terms) of the equation as the reference solution, and then consider how the second part (with perturbed terms) influences our answer. In general, perturbation theory solves such problem in two steps. First, we obtain the eigenfunctions and eigenvalues of the unperturbed Hamiltonian. Second, these eigenfunctions and eigenvalues are corrected to account for the perturbation's influence. As a matter of fact, perturbation theory gives these corrections as an infinite series of terms, which become increasingly smaller for well-behaved dynamic systems. Quite frequently, the corrections are only taken through first or second order. As long as the perturbation is small compared to the unperturbed potential or Hamiltonian, perturbation theory will tell us how to correct the solutions to the unperturbed problem and to approximately account for the influence of the perturbation. It has to be indicated that this theory has no direct relationship with our mathematical model, but it does serve as a stimulant to creative thought of our ODE's parameters estimation approach. Recently, we have presented a perturbation-based estimate algorithm for parameters of coupled ordinary differential equations and its application from chemical reactions to metabolic dynamics[45].

Perturbation search is a systematic direct search method for solving optimization problems that does not require any information about the gradient of the cost function. However, it does require the presence of pre-defined perturbed values for each parameter *p_i_*. Briefly speaking, the algorithm searches a set of symmetric points around the current parameter values, looking for the points where the cost function is lower than the cost function at the previous parameter set. This systematic direct search method can be used to solve a variety of parameters' estimation problems that are not well suited for standard optimization algorithms, including problems in which the cost function is discontinuous, nondifferentiable, stochastic or highly nonlinear.

While the gradient of cost functions is evaluated, the derivatives of the cost function can be intuitively known and simply formulated as 
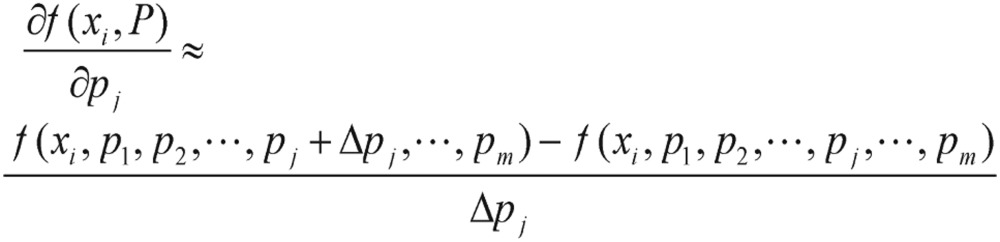
(4.1)

Thus, it is computed numerically through the finite difference method. The choice of Δ*p_j_* can be rather influential, and generally Δ*p_j_* should be taken as some fractional part of *p_j_*, such as 0.01 or 0.1.

Speaking of the gradient function or the “derivative” term in mathematics, we should also mention that, although the term of the gradient of cost function, such as the expression in the above equation, is not meaningful, it has already conveyed some sense of the “directional derivative”. In light of equation (31), it is also worthwhile to note that instinctive understanding of the principles of metabolic dynamics that govern how the trajectories or cost functions move in various situations is vital to success in parameters searching. As everyone knows, in calculus, the derivative is a measurement of how a function changes when the values of its inputs change. Loosely speaking, a derivative can be thought of as how much a quantity is changing at some given point. To interpret this term, a directional derivative, a partial derivative of *f* that measures its variation in the direction of the coordinate axes, should be introduced here, which enables us to find the rate of change of a function of two or more variables in any direction. For example, if *f* is a function of *x* and *y*, then its partial derivatives measure the variation in *f* in the *x* direction and the *y* direction. If we use three variables, we can define the directional derivatives in a similar manner. For instance, the directional derivative of *f* at (*x*_0_, *y*_0_, *z*_0_) in the direction of a unit vector 
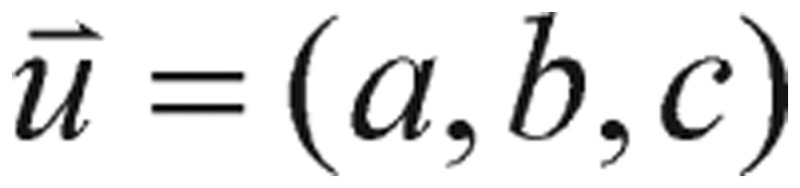
 is 
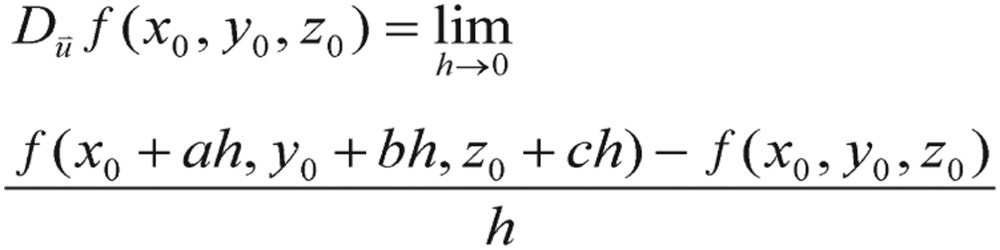
(4.2) if the limit exists. The directional derivative can be written as follows: 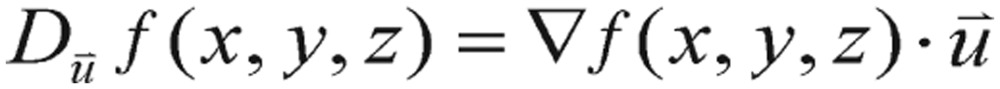
(4.3)

Now, suppose we have a function *f* of three variables, *x*, *y*, and *z*, and we consider all possible directional derivatives of *f* at a given point. These directional derivatives will provide us the rates of changes of *f* in all possible directions. The question is in which of these directions the function *f* changes the fastest and what the maximum rate of change is. According to equation (4.3), we have the answer: the maximum value of the directional derivative 
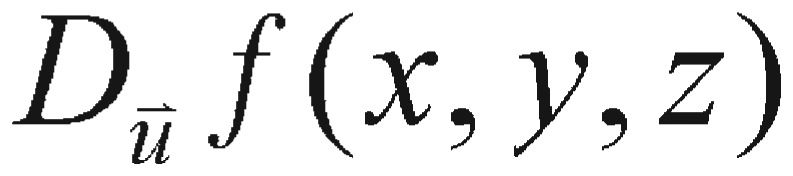
 is 
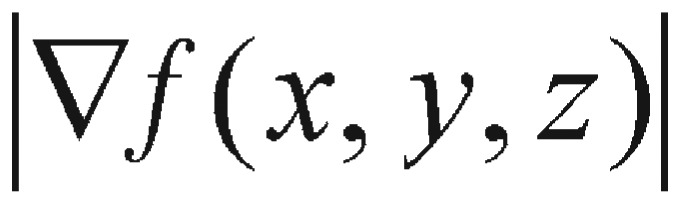
 and it only occurs when 
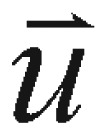
 is pointing to the same direction as the gradient vector 
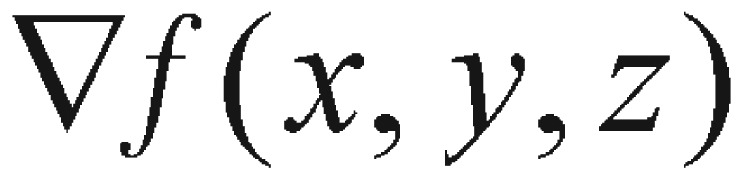
. A comparison between equation (4.1) and its similar equation (4.2) does enlighten us to pursue our perturbation search algorithm, even though the variables used in these two equations are different - one describes the variation of ODE parameter, and the other one represents the displacement on axis.

In numerical analysis, finite difference method does play a significant role. They are one of the simplest ways of approximating a differential operator and are extensively used in solving differential equations. The difference quotient is defined as the increment of the value of a function divided by the increment of the independent variable, which includes three common forms: forward, backward and central differences. A forward difference of function is an expression of the form *f*(*x+h*)-*f*(*x*). A backward difference of function uses the function values at *x* and *x*-*h*, instead of the values at *x*+*h* and *x*, which is *f*(*x*)-*f*(*x*-*h*). The central difference of function is given by *f*(*x+h/*2)-*f*(*x*-*h/*2). All those three function's increments divided by the independent variable's increment, *h*, will give three different approximated derivative values. The centered difference approach is more accurate than both forward and backward difference formulas. Conceptually, the central difference approach uses two points on both sides of *x*, so it can reach the 2^nd^ order accuracy if function *f* is expended in a Taylor series around the point *x*. It has to be noted here that this concept does give rise to motive ideas and becomes the source of inspiration for us to extend the perturbation theory idea to the estimation of ODE's parameters.

In fact, numerical optimization with the perturbation search method in the current study is a “trial and error” process. We begin with a single parameter as the starting example. Starting from an initial set of parameter values for which the initial cost function is known, a new set of parameter values is calculated by perturbing each of the initial parameter values, and the corresponding cost functions will be evaluated. More specifically, three trials, two perturbation trials plus one trial without perturbation, for each iteration run are simulated by choosing the algebraic sign (-), reversing of sign (+) and no sign of each base parameter values. The two computed cost function values *SSR*(*p-Δp*) and *SSR*(*p*+*Δp*), corresponding to negative and positive perturbations, plus the unperturbed cost function *SSR*(*p*) value are compared to the cost function value at the initial point or previous point. If a cost function value is less than the previous minimum cost function value, *SSR_min_*, the current set of parameter values for which the cost function has its least value becomes the new starting point for the next trial. If neither value is less than the previous *SSR_min_* value, the previous parameter values and its previous *SSR_min_* will remain the same for the next trial.

The multiplication rule can be used to determine the total number of outcomes in a sequence of events. In a sequence of *n* events, the first one has *k_1_* possibilities and the second event has *k_2_* and the third has *k_3_*, and so forth. Thus, the total number of possibilities of the sequence will be the product of all possibility values, *k_1_*·*k_2_*·*k_3_* …….

**Fig. 3:**
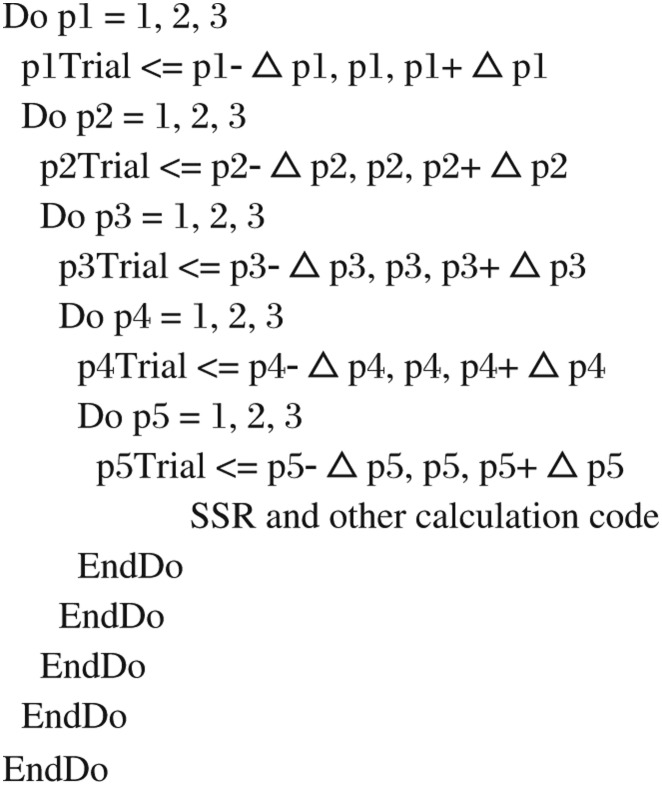
The algorithm for looping all five parameters.

It can be concluded that the 2-possibility *n* events produce a total of 2*^n^* possibilities. Similarly, if there is a sequence of *n* = 5 events which have three possibilities for each, it will yield the total possibility number 3*^n^* = 3^5^ = 243. The programming logic of this iterative parameter combination scheme is illustrated as the pseudo-code of 5-parameter-loop in ***Fig. 3***.

It is worthwhile to mention that the parameter optimization can also be performed by a systematic reducing parameter search range. For instance, the relatively-wide initial search region is selected and used for running a number of iterations until the cost function reaches a constant-like value. Then, a reduction in the size of the search region at subsequent iteration will be carried out. The next iterations will follow the same procedures except that the search ranges of parameters are smaller. Basically, we hope the accuracy of the parameters will be enhanced through this range-reducing approach. From the practical point of view, the range reduction factor can be proposed as any appropriate expression based on the results of a trial run and output of each iteration. These steps are repeated until almost no variation of the estimated parameters or any predefined convergent criteria of the parameters and cost function are met. However, this adjusted approach of parameter range reduction can be implemented differently to suit the properties each individual ODE system.

### Multiple-Shooting Method

In the initial-value approach of the ODE system, initial guesses for equation parameters are chosen and the dynamical equations are solved numerically. The parameters can be identified as those minimizing the cost function. For many dynamical simulations, this approach is numerically unstable by yielding a diverging trajectory or stopping in a local minimum. This problem can be circumvented by a multiple-shooting algorithm developed by Bock[41]–[44]. The basic idea of the algorithm is to consider the task as a multi-point boundary value problem. The fitting time interval [*t_i_, t_f_*] is partitioned into *k* subintervals, 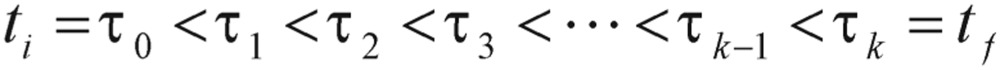
(5.4)

For each subinterval, local initial values are introduced as additional parameters. The dynamical equation is integrated piecewise and cost function is evaluated and minimized as exact in the initial value approach. While the dynamical parameter vector *P* is constant over the entire interval, the local initial values are optimized separately in each subinterval. Starting guesses for them are appropriately chosen to match the observations. This approach leads to an initially discontinuous trajectory, but is close to the measurements. The final trajectory will eventually be continuous as the computed solution at the end of one subinterval matches the local initial values of the next one. This condition is taken into account as equality constraints in the optimization procedure.

**Fig. 4:**
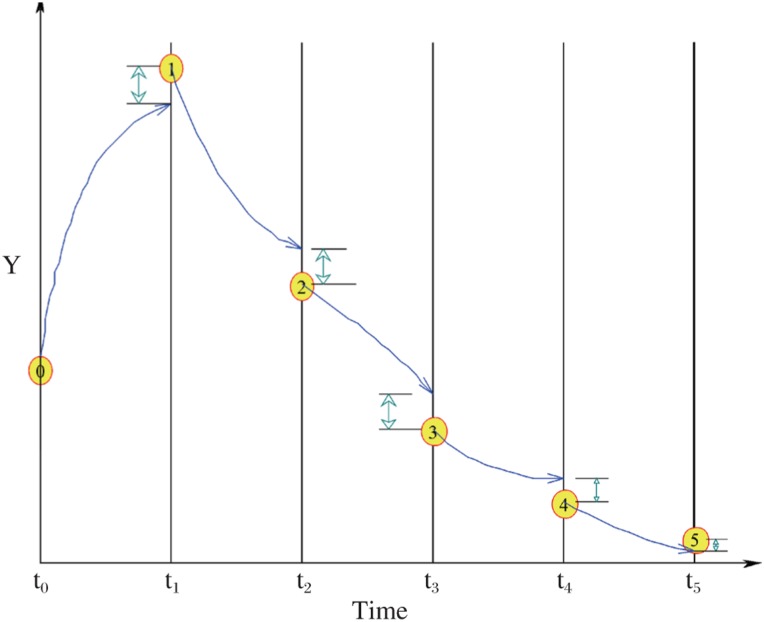
Sketchy diagram illustrates the application of multiple shooting method. The red circles with yellow-color fillings are the experimental sample points. The blue curve lines show the numerical integrated trajectories *y_i_*(*t_i_*, *y_i_*_-*1*_, *p*). The green lines with double-end arrows represent the errors between theoretical integrated values and experimental data values.

To achieve a good estimating of the domains of ODE parameters, we propose an optimal search with subinterval constraints and developed an algorithm to enforce it. The searched time interval is divided into a number of subintervals, which is the number of data points minus one. We have extended Bock's concept by replacing the one single-shooting SSR value in the initial-value approach by a summed value of “multiple” subintervals' SSR values. To illustrate the application of this method, a conceptual multiple-shooting method diagram is shown in ***Fig. 4***. As you can see from this figure, we sum over all the SSR components, which are represented as green lines with double arrows, of each subinterval.

### Coupled Ordinary Differential Equations Solver

To illustrate the solving method of ODE system, a system of first-order ODE is expressed as 
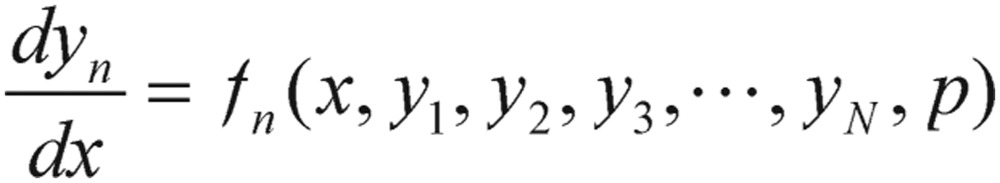
(6.1) where *n* = 1, 2, 3, ……, *N*

In the dynamics of time series, the independent variable *x* is usually replaced with time *t*. Therefore, a simple but general vector form of a system ODEs with the initial condition is formulated as 
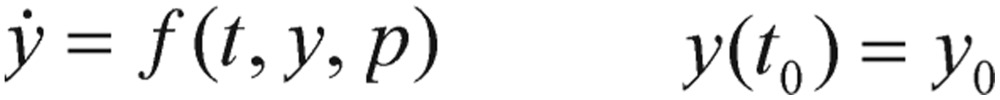
(6.2)

where *y* is the state vector of the system, *t* is time and *θ* = [*p*, *y*_0_] is a parameter vector consisting of all *p* parameters appearing in the right hand side of the ODE system and all initial-value vector *y_0_*.

Among numerical integration methods, the Euler method is the simplest integration method for solving ordinary differential equations, which is also called first-order Runge-Kutta method or Taylor series expansion of order one. The next common integration method is the second-order Runge-Kutta method, and its truncation error is higher than the one in the Euler method. However, detailed formulations and their applications of these methods are easily found in the literature and many numerical method books, which can also be clearly referenced in References[45],[46].

### Program flowchart

**Fig. 5:**
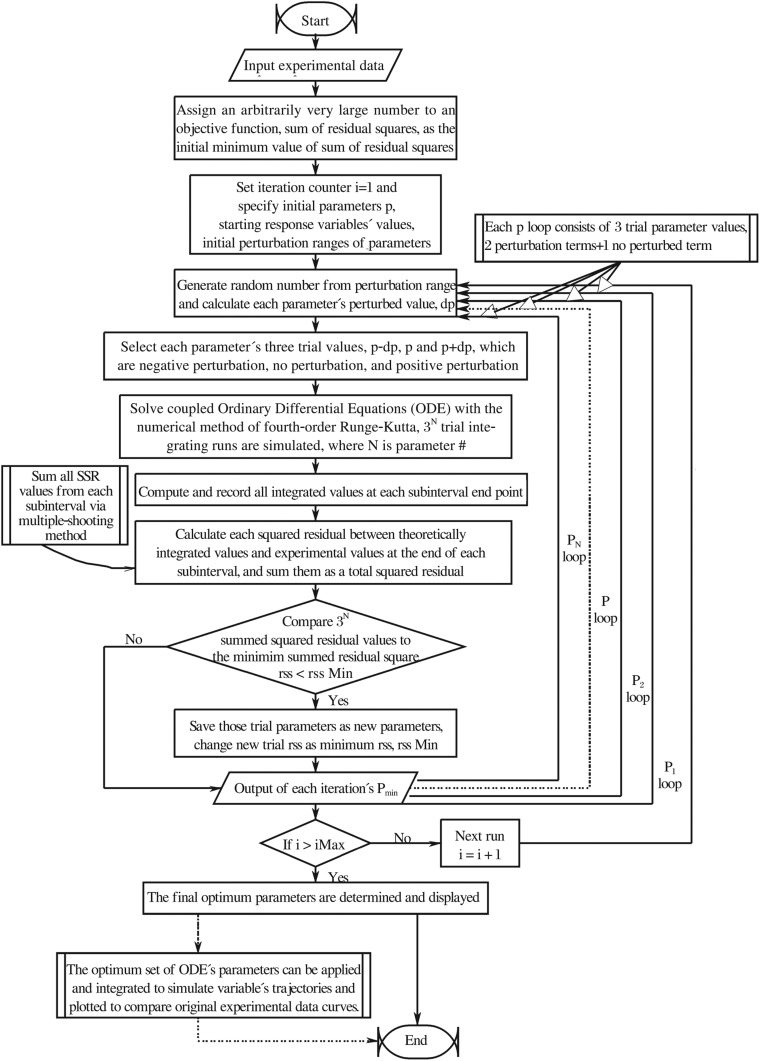
The flowchart of the current simulation program.

The flowchart of our numerical optimization algorithm for ODE's parameter estimations is presented in ***Fig. 5***. This flowchart outlines the order in which these functional elements in our computational approach were sequentially performed.

### Program requirements and specifications

The implementation of the computerized perturbation search methodology is straightforward. However, two functions or subroutines are required to accomplish the entire simulation. The first program is a coupled differential equation solver using any numerical integration method, such as the Runge-Kutta method. The second program is a random number generator. Fortunately, these two programs are available and easily found in most programming languages and scientific software packages. It should be mentioned that R is a free software environment for statistical computing and graphics, which provides a wide variety of statistical linear and nonlinear modeling. Therefore, our code of simulation model was written in R, but other languages, such as Fortran, C/C++, Java and Visual Basic, or other commercial mathematical and statistical software packages, such as Matlab^®^ and SAS^®^, could also be used. In addition, the hardware platform is IBM-compatible personal computer (PC) with Microsoft^®^ Windows 2000 and XP operating systems.

## RESULTS

Here we give an example with four sets of data points to depict the glucose-insulin regulatory dynamic trajectories[47]. ***Table 1*** shows an example for two subjects whose blood glucose and plasma insulin deviation values were sampled at four time points, initial-0 h, 1 h, 2 h, 3 h.

**Fig. 6:**
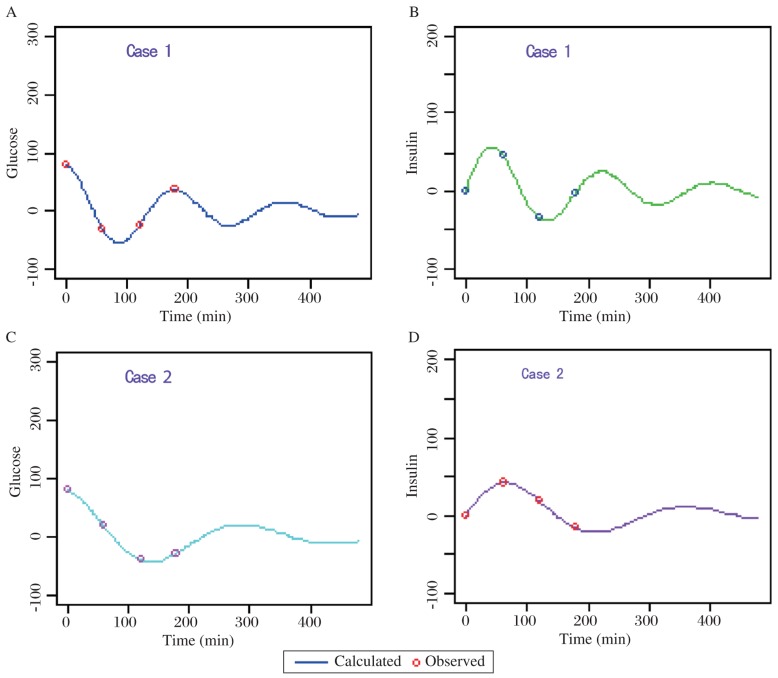
Simulations of the evolution of blood glucose and insulin concentration. A: blood glucose level of case 1. B: insulin level of case 2. C: blood glucose level of case 2. D: insulin level of case 2. In case 1, typical normal subject's parameter values are (p1 = 0.00437, p2 = 0.04016, p3 = 0.02989, p4 = 0.00450). In case 2, typical diabetic subject's parameter values are (p1 = 0.00439, p2 = 0.02996, p3 = 0.01506, p4 = 0.00453).

Based on those data, we estimated the four parameters, *p_1_, p_2_, p_3_*, and *p_4_*, by using our perturbation search algorithm and multiple-shooting method. In addition, those computed parameter values are also applied to make fitted curves compared to the original sample data points (see the circles in ***Fig.6***). As you can see from this figure, the theoretically simulated curves perfectly match the experimental data points.

As you can see from the graphs and the computed results, the four parameter values, which vary from person to person, determine the evolution of the trajectory over time. Both subjects started with the positive glucose variation 80 mg/dL (i.e., 80 mg/dL above the fasting baseline), which were immediately measured right after the ingestion of a large dose of glucose. As you can tell from those glucose curves, there is a rapid drop at 1 h time for case 1; in contrast, there is a relatively slower decrease for case 2. As a whole, the glucose trajectory of case 1 oscillates faster than that of the case 2, and it also quickly reaches the equilibrium state (i.e., fasting level). For the same reason, a similar faster variation of the plasma insulin concentration for case 1 produces quicker fluctuations (i.e., higher frequency). This reflects that case 1 has a shorter period of time on recovering from an initially large glucose excursion to an equilibrium fasting level. This result thus indicates that case 1 is relatively normal (i.e., non-diabetic) compared to case 2.

The R-based code fragments and outputs are listed below for your reference.

p1 <- 0.00437 p2 <- 0.04016 p3 <- 0.02989 p4 <- 0.00450 omega01 <- p2×p3 + p1×p4 alpha1 <- ( (p1 + p4) / 2 ) ˆ 2

p1 <- 0.00439 p2 <- 0.02996 p3 <- 0.01506 p4 <- 0.00453 omega02 <- p2×p3 + p1×p4 alpha2 <- ( (p1 + p4) / 2 ) ˆ 2

> omega01 [1] 0.001220047 > alpha1 [1] 1.966922e-05 > omega02 [1] 0.0004710843 > alpha2 [1] 1.98916e-05

Our computed oscillation periods are T_subject1_=179.88 min and T_subject2_=289.51 min. In Jorge Cruz's paper[47], their calculated oscillation periods are T_subject1_=179.9 min and T_subject2_=289.9 min. These extremely close (i.e., consistent) results imply that the first subject is normal and the second one is diabetic. This also indicates that our computation method is correctly validated.

As you can see from these R-based programming calculated results, the values of alpha (α) are less than the values of omega zero (ω_0_), so the oscillations types we have demonstrated are categorized as the under-damping type, α^2^ < *ω*_0_^2^. However, the detailed damping “type” classification is referenced in our study note.

**Table 1:** Four measurements (deviations from baselines) of blood glucose and plasma insulin concentration in two subjects.

Case	Variable	Initial	1 h later	2 h later	3 h later
1	Glucose	80	-29.7	-24.8	35.8
1	Insulin	0	46.4	-34.5	-1.5
2	Glucose	80	18.1	-38.8	-28.0
2	Insulin	0	41.4	18.6	-15.9

**Fig. 7:**
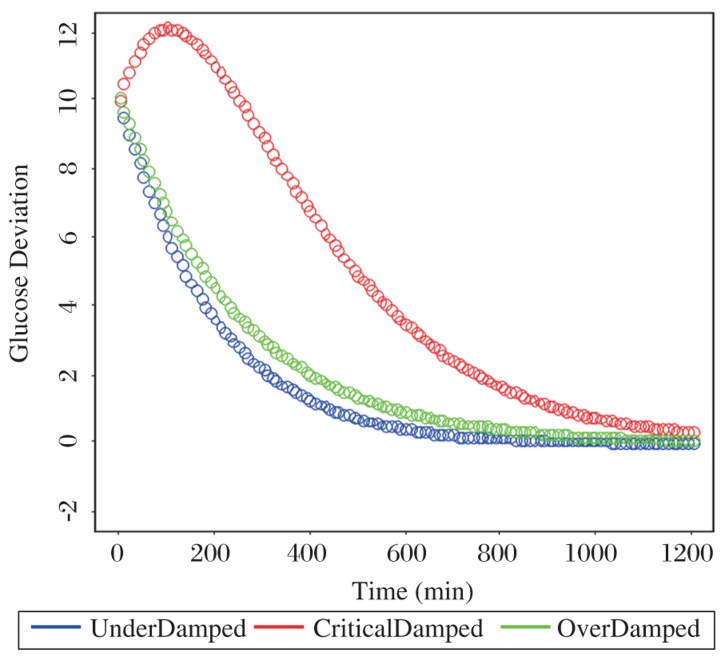
Examples of damped oscillatory motion with arbitrary constants ω=0.001 and α=0.005.

**Fig. 8:**
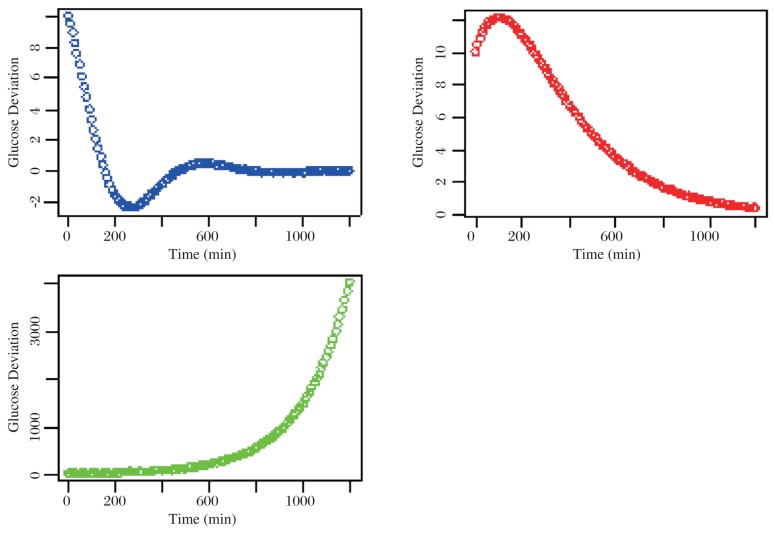
Examples of damped oscillatory motion with arbitrary constants ω=0.01 and α=0.005. The blue, red and green curves represent the under-damping, critical-damping and over-damping trajectories, respectively.

Based on the final solutions of the three-type glucose variation, those curves are plotted and compared. The R-based code fragments and graphical outputs are shown in the following two diagrams, ***Fig.7 and Fig.8***.

1^st^ case

c1 <- 10 c2 <- 0.1 omega <- 0.001 alpha <- 0.005 t <- seq(0, 1200, 10) gUnder <- exp(-alpha×t)×(c1×cos(omega×t)+c2×sin(omega×t)) gCritical <- (c1+c2×t)×exp(-alpha×t) gOver <- c1×exp(-(alpha-omega)×t)+ c2×exp(-(alpha+omega)×t) plot(t, gUnder, col=“blue”, xlab=“Time”, ylab=“Glucose Deviation”, xlim=c(0, 1200), ylim=c(-2, 12)) points(t, gCritical, col=“red”) points(t, gOver, col=“green”) legend(“topright”, c(“UnderDamped”, “CriticalDamped”, “OverDamped”), col=c(“blue”,“red”,“green”), lty = c(1, 1, 1), text.col=“purple”, bg=‘gray90’)

2^nd^ case

par(mfrow=c(2, 2)) c1 <- 10 c2 <- 0.1 omega <- 0.01 alpha <- 0.005 t <- seq(0, 1200, 10) gUnder <- exp(-alpha*t)*(c1*cos(omega*t)+c2*sin(omega*t)) gCritical <- (c1+c2*t)*exp(-alpha*t) gOver <- c1*exp(-(alpha-omega)*t)+ c2*exp(-(alpha+omega)*t) plot(t, gUnder, col=“blue”, xlab=“Time”, ylab=“Glucose Deviation”) plot(t, gCritical, col=“red”, xlab=“Time”, ylab=“Glucose Deviation”) plot(t, gOver, col=“green”, xlab=“Time”, ylab=“Glucose Deviation”)

The choices of two coefficients of the solutions, c_1_=10 and c_2_=0.1, are arbitrarily selected, but there is only one condition that needs to be met in this simulation - all the simulated data should be started at the almost same initial point, such as 10 in these plots. Thus, based on the same starting point, we can visualize and see the difference on how those metabolic trajectories move.

As you can see from ***Fig. 8***, the over-damping oscillation represented by a green curve is dramatically elevated to an unrealistic high level. In practice, this situation in plasma glucose response to GTT actually can never occur. On the other hand, we should suggest that the curve patterns in both the under-damping and critical-damping types could play a reasonable role on the platform of glucose metabolic simulations.

## DISCUSSION

Monitoring blood glucose concentration is crucial to the successful diagnosis and treatment of people with diabetes. The earlier the diagnosis of the disease the better the chances of controlling it with insulin and helping the subject live longer. One simple test for diagnosing diabetes is GTT, where the subject ingests a large amount of glucose, and then has his or her blood monitored for about 4-6 h following the glucose administration. The computational model in the present work has investigated this diabetic GTT metabolic test one step further, with respect to the already published Ackerman[11],[12],[40], Bolie[9], Wu[39], and Cruz[47] models, although they have provided a widely-accepted tool for clinicians and researchers to evaluate the excursions of blood glucose levels and to diagnose the existence of diabetes.

In the section of Results, we present a motivating example from the online published articles[47] regarding the theoretical diagnosis of diabetes. The classification of the dynamical system's behaviors, such as oscillating with period of time, *T*, usually depends on some conditions or relations between these system's parameters, such as damping factor and angular frequency.

The most notable motion from our simulations and graphs is the under-damping oscillation, which is described by the product of the exponential term and sinusoidal cosine function. This product solution gradually decreases the amplitude of the oscillation until it reaches the equilibrium state (i.e., the term of “fasting state” or “baseline” in diabetes field). The exponential term acts as a boundary constraint for the amplitude of the sinusoidal function, resulting in a gradual decrease of oscillation. Another important concept from the graph is that the period of the oscillation does not change, even though the amplitude is constantly decreasing. Therefore, the period, T=2π/*ω_0_*, is the good index to be computed in this investigation and other studies. Other than that reason, based on other researchers' previous experience and evidence, experimental testing of the model showed that the parameter, 
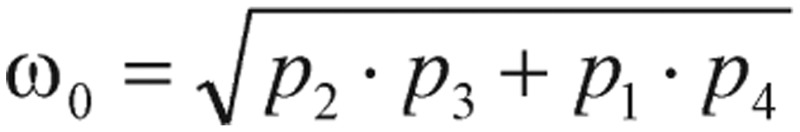
, was quite robust and proved to be a good indicator of diabetes. In particular, healthy individuals satisfied the criteria: 
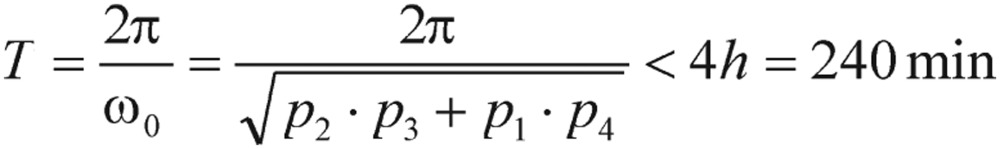
(7.1)

To simply interpret this mathematical expression, the blood glucose concentration of a non-diabetic who has just absorbed a large amount of glucose will be at or below fasting level in 4 h or less. On the contrary, the reversed inequality indicates diabetic case.


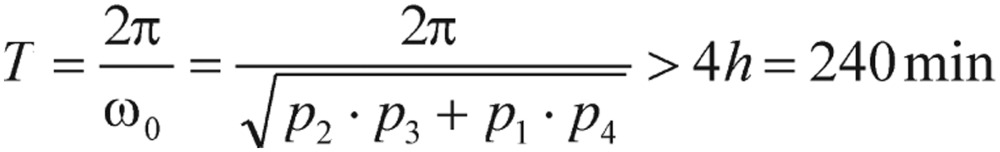
(7.2)

In practice, for a particular subject, the accuracy of the diagnosis can be benefited from multiple (i.e., repeated) measurements as the reliable ranges of the parameters, the values of ‘mean±standard deviation’ or even confidence intervals, can be accurately estimated. Thus, clinicians can safely evaluate the diagnostic conditions based on the reliably estimated parameters and the above formulas' criteria. However, due to the constraints of limited resources and the uneasy-fitted scheduled procedure time, GTT is usually performed only once within several months or a year. Nevertheless, our non-repeated GTT calculated result suggests that the dynamic response of diabetic subjects to typical hourly glycemic challenges extends over a much broader range in time domain than that of non-diabetic individuals. In other words, the oscillation period is higher in diabetic subject and lower in normal subject.

Ideal studies for characterizing blood glucose dynamics should include blood glucose measurements at a sufficiently regular sampling frequency to detect all variations over a period of several days (i.e., in MAGE evaluations), or even several weeks long (i.e., in LI measurements), while the subject is undergoing a wide range of activities, such as meals, exercise and sleep. However, our simulations on the GTT studies were based on an ideal case with only four sample points at the hourly time ranges, (0-3 h), which are not sufficient enough to fit them to an appropriate curve that describes the entire blood glucose dynamics as well as to examine the special features of glucose excursions. Theoretically speaking, there are at least eight well-recorded sample points to monitor patient's glucose regulatory phenomena.

Lastly, based on the above two equations as well as our calculated results in section Results clearly indicate that the Ackerman model and also the extended approach in the current study may play an important role in diagnosing diabetes, or even provide clinicians and scientists in endocrinology and metabolism fields insight into the transition nature of human's metabolic mechanism from normal to impaired glucose tolerance.

In conclusion, from a mathematical point of view, the conventional parameters estimation methods, such as the Gauss-Newton method, usually have the drawback of singular inverted matrix. The main purpose of our approach is to develop a simple, stable and formula-derivation-free computational program and, most importantly, to avoid divergence problem. Furthermore, there are no bounded constraints applied, and thus the movements of all equations' parameters during the entire computer simulation are absolutely free. Clinically speaking, in order to determine whether or not a subject has pre-diabetes or diabetes, health care providers usually conduct a GTT and apply standard HOMA and QUICKI methods. The aim of this study is to explore how to interpret those laboratory data as well as enhance the Ackerman mathematical model and how numerical analysis and computational iteration program are developed to search and identify coupled ODEs' parameter values.As presented in four recently published articles[45],[48]–[50] the proposed mathematical models and computation methods were successfully applied to have effectively direct measurements of insulin hormones secretion and dynamics in glucose metabolism. The present work addressed the application of multiple-shooting method and perturbation-search algorithm to biomedical problems, particularly to endocrinology and metabolism fields, which involves two coupled differential equations with four parameters describing the glucose-insulin regulatory system. Our demonstration showed that our approach could be used to practically evaluate the subject's glucose deviations and diagnose the diabetic disease as well as to theoretically serve as a promising starting point on ODE's parameters estimation. Hopefully, the current computer simulation can be studied further to provide investigators an effective tool and key information for the design of clinical studies that involve blood glucose dynamics and also for the possible development of new glucose monitoring systems. On the other hand, the extended and enhanced approach in the current study could play an important role in diagnosing diabetes, or even provide clinicians and scientists in endocrinology and metabolism fields insight into the transition nature of human metabolic mechanism from normal to impaired glucose tolerance. Furthermore, the mathematical models can be used to suggest better treatments, such as to improve scheduling of insulin injections, to regulate patient's diet, and have a solid pancreas or novel islet-cell transplant treatment. Due to both new experimental clinical studies and improved mathematical models, future treatments will certainly evolve to better regulate this metabolic disease's problem in diabetic patients. Lastly, it should be emphasized that our integrated approach of perturbation search and multiple-shooting methods provides an attractive feature to numerical estimation of the coupled differential equations, although parameters estimation has been recognized as a well-known challenge and accomplishment in mathematics and statistics.
